# Factors influencing the use of epidural labor analgesia: a cross-sectional survey analysis

**DOI:** 10.3389/fmed.2023.1280342

**Published:** 2024-02-07

**Authors:** Wei Li, Na Wu, Shuangqiong Zhou, Weijia Du, Zhendong Xu, Zhiqiang Liu

**Affiliations:** ^1^Department of Anesthesiology, Shanghai Key Laboratory of Maternal-Fetal Medicine, Shanghai Institute of Maternal-Fetal Medicine and Gynecologic Oncology, Shanghai First Maternity and Infant Hospital, School of Medicine, Tongji University, Shanghai, China; ^2^Nursing Department of Shanghai Key Laboratory of Maternal-Fetal Medicine, Shanghai Institute of Maternal-Fetal Medicine, and Gynecologic Oncology, Shanghai First Maternity and Infant Hospital, School of Medicine, Tongji University, Shanghai, China

**Keywords:** epidural labor analgesia, labor pain, personal factors, organizational factors, vaginal delivery

## Abstract

**Introduction:**

This study aimed to explore the personal and organizational factors influencing the lack of implementation of epidural labor analgesia (ELA).

**Methods:**

This study was conducted at the Shanghai First Maternity and Infant Hospital, School of Medicine, Tongji University, Shanghai, China. A total of 451 women who underwent vaginal delivery without ELA between 8 October 2021 and 30 March 2022, were included. A questionnaire was used to collect the relevant data. We derived and validated the variable, without ELA, by using binary logistic regression analysis.

**Results:**

Of the total 451 included, 355 (78.7%) initially preferred ELA, whereas 96 (21.3%) rejected it directly. Five variables were validated (*p* < 0.05): multiparas, ELA would lead to back pain, experienced ELA in previous delivery, the inner attitude toward labor pain, and blood routine and coagulation function not being tested within 14 days. The sensitivity and specificity of this model were 96.3 and 69.8%, respectively.

**Conclusion:**

The corresponding training should be provided to the medical staff to identify women at high risk of rejecting ELA during the prenatal examination process using a questionnaire, then provide them with knowledge regarding ELA, so that ELA can benefit more mothers. Additionally, the existing organizational factor should be addressed in order to efficiently provide ELA services to mothers.

**Clinical trial registration:**

This study was registered at the Chinese Clinical Trial Registry (Chi CTR 2000034625) on July 12, 2020

## 1 Introduction

Labor pain is the worst pain that most women experience in their lives (1). With the development of medical technology and the progress of concepts, epidural labor analgesia (ELA) has been popular since the 1980s in Western developed nations; current ELA rates are as high as 80–90% (1, 2). ELA has been recognized as the gold standard in labor analgesia (3–7). Acute labor pain may lead to adverse effects on functional, social, and psychological well-being, including postpartum depression (8). A lack of pain management is perceived as a lack of humanitarian spirit.

In our unit since 2013, one anesthesiologist per shift has been on duty, allowing an anesthesiologist to be available 24 h, and the rate of ELA has reached 80–85% of cases. Although the advantages of ELA are clear, some parturients remain reluctant to accept it. The prevalence and influencing factors of no ELA management are unclear. Assessing these influencing factors could help distinguish women inclined to reject ELA management and the organizational factors leading to this situation. We hypothesized that the absence of ELA management was associated with individual and management disparities in our unit. This study was novel because this was the first time a binary logistic regression model was used to explore the relevant personal and organizational factors influencing parturients who underwent labor without ELA.

## 2 Methods

### 2.1 Ethics statement

This study was approved by the ethics committee of the Shanghai First Maternity and Infant Hospital (approval code: KS 20204, Chairperson Ye Luo) on 11 June 2020, and was registered at the Chinese Clinical Trial Registry (Chi CTR 2000034625) on 12 July 2020. Written informed consent for publication of their details was obtained from the study participants.

### 2.2 Study population

This prospective cohort study was conducted at the Shanghai First Maternity and Infant Hospital. The inclusion criterion was parturients who delivered without ELA and a total of 509 women were included. The inclusion criterion was parturients who delivered without ELA. The exclusion criteria were: (1) women with contraindications to epidural puncture; (2) women who were aged less than 18 years, with stillbirth, or with physical or mental disabilities; (3) women who delivered out of the hospital or in the emergency ward; and (4) women with missing data for the interview.

Parturients who delivered without ELA from 8 October 2021 to 30 March 2022 were recruited and interviewed to complete the questionnaire. The participants were divided into the Requested and Rejected groups based on whether they received ELA.

Sample size calculation: the method is *post hoc* power analysis based on the actual studied sample size. As mentioned previously, the overall rate of labor without ELA is approximately 15%, so the *π* = 0.15, *n* = 451, specify *δ* = 0.035, U_α_* $\sqrt{\pi *\left(1-\pi \right)/n}$ =0.035, U_α_* $\sqrt{0.15*\left(1-0.15\right)/451}$ =0.035, U_α_ = 2.08, α<0.05.

Data were obtained from two sources: the medical records regarding whether ELA was used, prenatal care, and outcomes of labor; and interview with the questionnaire. The researchers interviewed the participants 1–3 days after delivery during their stay at the postpartum ward using a standardized questionnaire. This study only included those parturients who delivered vaginally in the delivery ward. ELA could be administered to parturients only if they requested it from the incubation period of labor to full cervical dilatation, without ELA contraindications. The questionnaire was prepared based on the clinical experience of anesthesiologists as well as some previous studies (9–11).

### 2.3 Outcome

We evaluated delivery without the use of ELA or with requested ELA in our delivery room as a binary composite variable. Parturients who used non-pharmacological methods of analgesia, including doula, liberal position, water birth, Lamaze breathing, transcutaneous electrical nerve stimulation, and music were included in the no ELA group unless their medical records specifically mentioned ELA placement.

### 2.4 Statistical analysis

All statistical analyses were performed using SPSS (version 26.0, SPSS Inc., Chicago, IL, USA). Gestational weeks were transformed into ordered multiclass variables (Term >37 W, late preterm 34 ~ 37 W, moderately preterm 32 ~ 34 W, very preterm 28 ~ 32 W, extremely preterm <28 W). Categorical variables were presented as percentages (%) and ranked data as rank averages.

Parturients were randomly divided into two sets: one for the derivation of significant prognostic factors and the other for validation of the prognostic model. For the derivation sets, we first identified factors related to ELA through univariate analyses by using the χ^2^ test and rank sum test when appropriate. Following this, all variables with a significance level less than 0.05 in the previous phase were analyzed by using binary logistic regression (Backward Wald test). Subsequently, all the variables with a *p* value less than 0.05 in the derivation set were further tested in the validation set using binary logistic regression (Backward Wald test). All analyses were two-tailed, and statistical significance was set at *p* < 0.05.

## 3 Results

**Figure 1:**
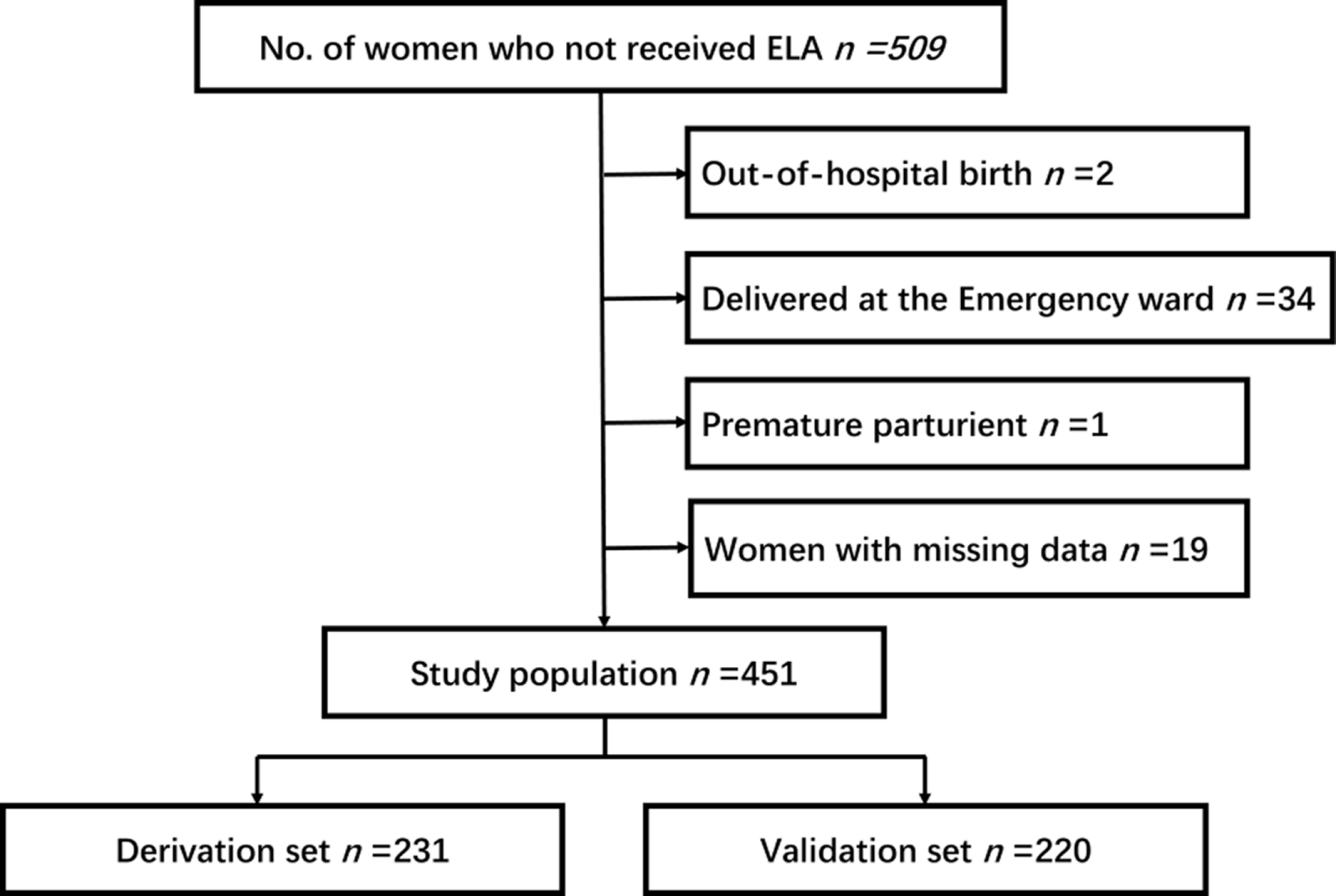
Flow diagram of included and excluded samples.

The 451 women included in this study who did not receive ELA were divided into two groups: those who rejected it directly (96, 21.3%) and those who requested it during labor (355, 78.7%) (Figure 1). The demographic characteristics of the two groups are presented in Table 1. There were no statistical differences in height, body weight, BMI, age, levels of education, nationality, and regions between the two groups. The incidences of multiparas (*p* = 0.00) and parturients who were not permanent residents of Shanghai (*p* = 0.00) in the rejected group were significantly higher than those in the requested group.

**Table 1:** Demographics of the parturients.

	Requested	Rejected	F/*χ*^2^	*p*
Height (cm)	162.08 ± 4.89	162.03 ± 5.13	0.00	0.91
Weight (kg)	67.45 ± 8.39	68.07 ± 8.68	0.41	0.56
BMI (kg/m^2^)			0.99	0.89
< 18	1 (0.3)	1 (1.0)		
18–24	101 (28.5)	27 (28.1)		
25–29	224 (63.1)	60 (62.5)		
>30	29 (8.2)	8 (8.3)		
Age (year)	31.46 ± 3.71	33.08 ± 3.99	0.16	0.69
Parity			35.77	0.00
Nulliparas	172 (48.5)	14 (14.6)		
Multiparas	183 (51.5)	82 (85.4)		
Permanent residence in Shanghai			17.62	0.00
Yes	354 (99.7)	90 (93.8)		
No	1 (0.3)	6 (6.3)		
Education			5.92	0.21
Middle school or less	15 (4.2)	9 (9.4)		
High school	28 (7.9)	11 (11.5)		
Junior college	56 (15.8)	16 (16.7)		
College	170 (47.9)	40 (41.7)		
postgraduate	86 (24.2)	20 (20.8)		
Nationality			0.76	0.38
Han nationality	341 (96.1)	94 (97.9)		
Minority nationalities	14 (3.9)	2 (2.1)		
Religions			1.67	0.64
Buddhism	5 (1.4)	0		
Christianity	3 (0.8)	1 (1.0)		
Islam	1 (0.3)	0		
None	345 (97.5)	95 (99.0)		

Values are presented as mean ± SD, median (range), or n (%).

Subsequently, the parturients’ knowledge of ELA was examined (Table 2). The results showed that compared with parturients in the requested group, large proportions of parturients in the rejected group were more concerned that ELA would slow down the progression of labor (*p* = 0.00), result in lower back pain (*p* = 0.00), damage the nervous system (*p* = 0.00), impair their own intelligence (*p* = 0.02), impair memory (*p* = 0.00), impair new-born breastfeeding (*p* = 0.01), or impair their newborn’s intelligence development (*p* = 0.00).

**Table 2:** Knowledge about ELA.

	Requested	Rejected	*χ* ^2^	*p*
ELA would slow the progression of labor			11.99	0.00
Right	57 (16.1)	21 (21.9)		
Wrong	150 (42.3)	22 (22.9)		
Unknown	148 (41.7)	53 (55.2)		
ELA would result in lower back pain			24.11	0.00
Right	69 (19.4)	36 (37.5)		
Wrong	161 (45.4)	19 (19.8)		
Unknown	125 (35.2)	41 (42.7)		
ELA would damage the nervous system			20.29	0.00
Right	49 (13.8)	28 (29.2)		
Wrong	190 (53.5)	29 (30.2)		
Unknown	116 (32.7)	39 (40.6)		
ELA would impair intelligence			8.28	0.02
Right	19 (5.4)	10 (10.4)		
Wrong	242 (68.2)	51 (53.1)		
Unknown	94 (26.5)	35 (36.5)		
ELA would impair memory			10.95	0.00
Right	44 (12.4)	24 (25.1)		
Wrong	211 (59.4)	43 (44.8)		
Unknown	100 (28.2)	29 (30.2)		
ELA would impair new-born breastfeeding			9.05	0.01
Right	22 (6.2)	6 (6.3)		
Wrong	237 (66.8)	49 (51.0)		
Unknow	96 (27.0)	41 (42.7)		
ELA would impair a newborn’s intelligence			14.69	0.00
Right	11 (3.1)	9 (9.4)		
Wrong	258 (72.7)	52 (54.2)		
Unknown	86 (24.2)	35 (36.5)		
ELA can provide analgesia or anesthesia for artificial dissection of the placenta, perineal laceration suture, shoulder dystocia, and cystotomy			5.25	0.07
Right	38 (10.7)	12 (12.5)		
Wrong	153 (43.1)	29 (30.2)		
Unknown	164 (46.2)	55 (57.3)		

Values are presented as n (%). ELA, epidural labor analgesia.

The participants were asked to rate the pain during labor using the *visual analog scale* (Table 3). The VAS of parturients in the rejected group was significantly lower than that in the requested group when the cervix was dilated to 2 cm (*p* = 0.00) and fully dilated (*p* = 0.00).

**Table 3:** Grades of pain experienced during labor.

	Rank average	H	*p*
The pain grade when the cervix dilated to 2 cm		16.24	0.00
Requested	237.96		
Rejected	181.78		
The pain grade when the cervix is fully dilated		10.27	0.00
Requested	234.72		
Rejected	193.74		

Values presented are rank average.

We asked about the possible reasons for not accepting ELA by themselves (Table 4). The incidence of being able to bear the labor pain in the rejected group was higher than that in the requested group (*p* = 0.00). When the requested group parturients asked for ELA, the incidence of the anesthesiologist being busy with other parturients was higher than that in the rejected group (*p* = 0.00). Compared with the requested group, the incidence of needle phobia in the rejected group was significantly higher (*p* = 0.01). Where there were regular blood routine and coagulation function tests 14 days before the requested ELA, we have replaced the patients’ self-reports with accurate data from medical history. There was no “family member opposed ELA,” so this factor was not shown in the table.

**Table 4:** Possible reasons for mothers not choosing ELA.

	Requested	Rejected	*χ* ^2^	*p*
Pain grade was middle-moderate, tolerable			62.93	0.00
Yes	2 (0.6)	19 (19.8)		
No	353 (99.4)	77 (80.2)		
Pain was severe and endurable			45.59	0.00
Yes	0 (0.00)	12 (12.5)		
No	355 (86.5)	84 (87.5)		
Cost of ELA			3.71	2.13
Yes	0 (0.0)	1 (1.0)		
No	355 (100.0)	95 (99.0)		
Anesthesiologist was busy			14.53	0.00
Yes	48 (13.5)	0 (0.0)		
No	307 (86.5)	96 (100.0)		
Prolapse of lumbar intervertebral disk			1.48	0.32
Yes	12	1		
No	343	95		
Thrombocytopenia in pregnancy			1.92	0.35
Yes	7 (2.0)	0 (0.0)		
No	348 (98.0)	96 (100.0)		
Subcutaneous injection of heparin			0.99	0.38
Yes	1 (0.3)	1 (1.0)		
No	354 (99.7)	95 (99.0)		
Lumbar spondylolisthesis			0.27	1.00
Yes	1 (0.3)	0 (0.0)		
No	354 (99.7)	96 (100.0)		
Scoliosis			0.27	1.00
Yes	1 (0.3)	0 (0.0)		
No	354 (99.7)	96 (100.0)		
Skin eczema			0.27	1.00
Yes	1 (0.3)	0 (0.0)		
No	354 (99.7)	96 (100.0)		
No confidence in ELA			3.71	2.13
Yes	0 (0.0)	1 (1.0)		
No	355 (100.0)	95 (99.0)		
Needle phobia			10.67	0.01
Yes	2 (0.6)	5 (5.2)		
No	353 (99.4)	91 (94.8)		
Blood routine and coagulation function not being tested within14 days			26.47	0.00
Yes	99 (27.9)	3 (3.1)		
No	256 (72.1)	93 (96.9)		
Novel coronavirus nucleic acid being tested			1.77	0.23
Yes	24 (6.8)	3 (3.1)		
No	331 (93.2)	93 (96.9)		
Premature birth			2.39	0.17
Yes	21 (5.9)	10 (10.4)		
No	334 (94.1)	86 (89.6)		
Multiparas, labor process is fast			2.99	0.11
Yes	3 (0.8)	3 (3.1)		
No	352 (99.2)	93 (96.9)		
Cervix near full dilation when entering the delivery ward			3.05	0.13
Yes	11 (3.1)	0 (0.0)		
No	344 (96.9)	96 (100.0)		

Values are presented as n (%).

We explored how the parturients gained knowledge of ELA (Table 5). Approximately half of the women in both groups learned about ELA by browsing websites and communicating with friends and relatives. However, the incidence of women who once had ELA in the requested group was significantly higher than that in the rejected group (*p* = 0.01). Other methods of acquaintance with ELA knowledge only accounted for a small proportion. We found that only a small proportion of pregnant women attended the hospital childbirth education classes or received the ELA brochure. Additionally, we noticed that the incidence of being “not familiar with ELA” in the rejected group was markedly higher than in the requested group (*p* = 0.00).

**Table 5:** Medium of obtaining ELA knowledge.

	Requested	Rejected	*χ* ^2^	*p*
Received ELA brochure			2.61	0.12
Yes	61 (17.2)	10 (10.4)		
No	294 (82.8)	86 (89.6)		
Network			1.16	0.28
Yes	203 (57.2)	49 (51.0)		
No	152 (42.8)	47 (49.0)		
Friends and relatives			1.54	0.21
Yes	199 (56.1)	47 (49.0)		
No	156 (43.9)	49 (51.0)		
Once ELA			7.66	0.01
Yes	92 (25.9)	12 (12.5)		
No	263 (74.1)	84 (87.5)		
Television programs			0.75	0.39
Yes	53 (14.9)	11 (11.5)		
No	302 (85.1)	85 (88.5)		
Newspapers and books			0.81	0.37
Yes	41 (11.5)	8 (8.3)		
No	314 (88.5)	88 (91.7)		
Childbirth education			0.68	0.41
Yes	40 (11.3)	8 (8.3)		
No	315 (88.7)	88 (91.7)		
Midwife			0.63	0.45
Yes	64 (18.0)	14 (14.6)		
No	291 (82.0)	82 (85.4)		
Obstetrician			0.97	0.32
Yes	59 (16.6)	12 (12.5)		
No	296 (83.4)	84 (87.5)		
Not familiar with ELA			21.24	0.00
Yes	13 (3.7)	16 (16.7)		
No	342 (96.3)	80 (83.3)		

Values are presented as n (%).

We investigated the choice of doula during labor and the attitudes toward pain of these parturients (Table 6). The incidence of doula being selected in the requested group was significantly higher than that in the rejected group (*p* = 0.00). Unexpectedly, the incidence of those who could calmly bear labor pain in the rejected group was significantly higher than that in the requested group; however, feeling nervous and fearful toward labor pain in the rejected group was significantly lower than that in the requested group (*p* = 0.00). Although the incidence of being willing to choose ELA for the next natural labor delivery in the rejected group was significantly lower than that in the requested group, nearly half of them stated they would still reject ELA and one-third of them were uncertain whether to use ELA for the next natural labor delivery. Only 3.1% of parturients in the requested group stated they would deny ELA for the next natural labor delivery.

**Table 6:** Choice of other analgesic methods during labor and attitudes toward pain.

	Requested	Rejected	*χ* ^2^	*p*
Selected Doula			18.23	0.00
Yes	243 (68.5)	43 (44.8)		
No	112 (31.5)	53 (55.2)		
Attitude toward labor pain			32.76	0.00
Calm	136 (38.3)	68 (70.8)		
Nervous	154 (43.4)	22 (22.9)		
Fearful	65 (18.3)	6 (6.3)		
Will you choose ELA for the next natural labor delivery?				
No	11 (3.1)	46 (47.9)	179.04	0.00
Yes	296 (83.4)	19 (19.8)		
Uncertain	48 (13.5)	31 (32.3)		

Values are presented as n (%).

The derivation and validation of factors for failure to use ELA are shown in Table 7. The sample was randomly divided into a derivation set and a validation set. All variables with *p* < 0.05 in Tables 1–7 were included in the binary regression analysis of the derivation set. Subsequently, all the variables with a *p* value less than 0.05 in the derivation set were further tested in the validation set using binary logistic regression (Backward Wald test). The validation set was not involved in variable selection in the stepwise binary logistic model. Five of eight variables were validated (*p* < 0.05): multiparas, ELA would lead to back pain, experienced ELA in previous delivery, the inner attitude toward labor pain, and blood routine and coagulation function not being tested within 14 days prior to applying for ELA.

**Table 7:** Factors associated with no ELA: binary logistic regression analysis.

A. Omnibus tests of the model coefficients				
		*χ* ^2^	df	*p*
Step 27	Step	10.55	2	0.00
	Block	126.76	20	0.00
	Model	126.76	20	0.00

B. Hosmer and lemeshow test			
Step	*χ* ^2^	df	*p*
27	4.22	8	0.84

C. Classification table					
Observed			Predicted		
			ELA		Percentage correct
			Yes	No	
Step 27	ELA	Yes No	181	7	96.3
			13	20	69.8
	Overall percentage				91.3

D. Variables in the equation						
Significant variables in univariate analysis	Binary logistic regression					
	Derivation set (*n* = 231)			Validation set (*n* = 220)		
	*p*	OR	95% CI	*p*	OR	95% CI
Multiparas	0.00	13.34	3.26–54.50	0.00	10.20	3.89–26.76
ELA will cause back pain	0.01	—	—	0.01	—	—
ELA will cause back pain (1)	0.37	2.24	0.39–13.04	0.01	3.74	1.48–9.47
ELA will cause back pain (2)	0.01	10.26	1.93–54.61	0.00	5.69	2.10–15.43
Previously used ELA	0.00	12.35	2.26–67.68	0.00	4.11	1.56–10.86
Inner attitude toward ELA	0.01	—	—	0.01	—	—
Inner attitude toward ELA (1)	0.02	13.67	1.42–131.55	0.07	3.43	0.92–12.71
Inner attitude toward ELA (2)	0.56	1.97	0.20–19.33	1.00	1.00	0.25–3.96
Blood routine and coagulation function reports within 14 days	0.03	2.21	1.09–4.50	0.00	2.96	1.51–5.79
Pain grade was middle-moderate, tolerable	0.04	0.02	0.00–0.85			
ELA would impair new-born breastfeeding	0.00	—	—			
ELA would impair new-born breastfeeding	0.00	19.35	3.88–96.55			
ELA would impair new-born breastfeeding	0.65	1.77	0.15–20.90			

## 4 Discussion

### 4.1 Main findings

To the best of our knowledge, this is the first time our unit has investigated the influencing factors for rejecting ELA. Surprisingly, the percentage of women in the rejected group was only about 21% of those who did not receive ELA. Binary logistic regression results show that personal factors included multiparas, concern that ELA would lead to back pain, experienced ELA in previous delivery, and the inner attitude toward labor pain. The organizational factor was parturients whose regular blood routine and coagulation function had not been tested within 14 days, would be rejected by the anesthesiologist.

### 4.2 Strengths and limitations

Our unit is a tertiary maternity and infant hospital and based on our records, we have been implementing ELA since 2000. However, it was not until 2018 that the National Health Commission began to advocate ELA in secondary general hospitals and maternity hospitals for mothers throughout the nation. Moreover, the anesthesiologist in our unit is on duty 24 h, so theoretically we can implement ELA in time for paturients. The rate of ELA in our unit has reached 80–85%, reaching the level of Western developed countries. For women who did not adopt ELA, we interviewed them face to face in the delivery room or ward after delivery to effectively collect relevant information. After preliminary analysis, maternal patients who did not adopt ELA were divided into the “Request group” and “Reject group” and binary logistic regression analysis was used to explore possible organizational and individual factors affecting the choice to request or reject ELA.

We originally intended to conduct a multicenter survey study. However, we were unable to do so, due to the length of time ELA has been available in these general hospitals was short, different policies and conditions for ELA in different hospitals. According to a survey in China, the average ELA rate was only 17.3% in 2015 (12). This study is a retrospective study, and the physical and mental state of postpartum women who did not use ELA for natural delivery may affect the authenticity and accuracy of the survey. As this study comes from a single center, compared with multicenter surveys, the sample size is not large enough, and the results may have certain limitations. Even so, this conclusion still holds significance for secondary-level hospitals.

### 4.3 Comments

The intensity of pain is roughly proportional to the extent of cervical dilation during the first stage of labor, and inversely proportional to the duration of the interval between uterine contractions (13). The expectation of the intensity of pain in labor and one’s attitude toward pain largely determines whether one adopts ELA. If women have low expectations about the intensity of pain in labor, have a positive attitude toward the impending childbirth, prefer vaginal delivery, express positive attitudes toward pain management without medication, and had a high manifestation of a sense of security, they are more inclined to not use ELA (14, 15). Unlike a 2015 study in France (11), this study did not find that under unfavorable social conditions mothers prefer to reject ELA. Moreover, there was no significant difference in the educational composition between the two groups.

Inconsistent with the same French study, (11) we also found that multiparous women were more likely to reject ELA. This result can be explained by multipara having previously experienced labor pain, with psychological expectations of pain degree, or with pain considered lighter than primiparous pain, and can face it calmly, or have a shorter average duration of labor. Moreover, compared with primiparas, multiparas can easily express their preference for ELA and participate more actively in decision-making (16, 17).

However, multiparity and advanced cervical dilation are not always related to shorter labor (18). In addition to the non-pharmacological methods Lamaze breathing and doula which can be offered to women admitted in advanced labor, remifentanil, a pharmacological method, pumped intravenously can effectively and safely relieve labor pain (19). Intravenous remifentanil patient-controlled analgesia as an alternative to ELA for vaginal delivery has gained widespread recognition (20, 21).

A decade ago, several surveys indicated that most women thought that ELA would result in permanent backache (22–24). However, a systematic review of studies demonstrated that EA has no impact on the risk of C-section, instrumental vaginal delivery for dystocia, long-term backache, breastfeeding, or neonatal Apgar scores (25).

Research has shown a significant correlation between maternal education level and understanding of ELA (26). Thanks to the significant role played by the internet in the dissemination of ELA knowledge in recent years, we have not found this trend. However, the results of this study indicated that pregnant women believe more in their own experience of ELA. An interesting trend that can be described is that the parturients who selected ELA will select it again, while the parturients who rejected ELA will reject it again. Similar trends were found in another study (10). As aforementioned, in 2018, the National Health Commission began to advocate ELA throughout the nation, and our unit began to administer ELA in 2000. Parturients who are permanent residents in Shanghai or live in other cities may not be well acquainted with ELA and they still had many misunderstandings and concerns. Consequently, we found that nearly 30% of parturients in the rejected group would rather endure psychological tension or fear than use ELA to reduce labor pain. They perhaps thought that being a mother meant to endure pain.

Surprisingly, although there was no statistical difference in the perception of ELA, level of education, and access to ELA knowledge between Shanghai parturients and outsiders, those women who lived in other cities and only came to our unit for prenatal examination and delivery were at a higher risk of refusing ELA. They perhaps deliberately provided incorrect information when filling out the questionnaire.

A meta-analysis showed that approximately 20–30% of young adults have a needle phobia and there is a higher prevalence in women than in men (27). To our surprise, this study showed that approximately 6% of pregnant women refused ELA due to a needle phobia and preferred to endure labor pain. However, they may be able to overcome their fear and choose ELA rationally. In the past, this psychological factor was ignored and should be taken into consideration in further studies by providing counseling for this high-risk group.

A recent study suggests that awareness and fear of ELA were moderately correlated, (28) the less the fear, the greater the possibility of opting for ELA (2, 10, 29). This study shows that browsing websites and communicating with relatives and friends who have already used ELA were the main ways to acquire ELA knowledge, with only a few women wanting to learn about ELA by attending the pregnant women’s classes held in our unit. However, the knowledge spread by non-professional websites and friends and relatives may lead to a misconception and fear of ELA. An example of this was that pregnant women were concerned that ELA would damage neonatal intellectual development (30) Therefore, our unit should produce an official website to fully educate the women and provide high-quality labor analgesia services to eliminate maternal misunderstanding and fears of labor analgesia.

Although maternal platelet levels as low as 70–75 × 10^9^/L are considered the safe boundary to perform ELA (3), approximately 12% of pregnant women meet the diagnosis of thrombocytopenia during pregnancy (31). Some women, because of autoimmune diseases, anti-phospholipid antibody syndrome, or systemic lupus erythematosus, need subcutaneous injections of low molecular weight heparin (LMWH) for anticoagulation and fetal protection. Thus, our unit stipulates that the normal blood routine and coagulation function should be reported within 2 weeks prior to performing an epidural puncture. If parturients inject LMWH for thromboprophylaxis, ELA should be implemented at least 10–12 h after the LMWH dose; if parturients receive treatment doses of LMWH, at least 24 h is recommended to ensure normal hemostasis at the time of ELA (32). Therefore, if a parturient is admitted to the emergency department, and the labor process is progressing too fast without blood routine and coagulation function reports, then it may be too late to perform ELA. Another organizational factor why a proportion of mothers in the requested group did not receive ELA is that we only have one anesthesiologist on duty per shift, so he can only give EP to the mothers one by one. However, it is often a group of mothers who request EP almost simultaneously. Due to the shortage of anesthesiologists, some of the “request group” mothers were unable to receive EP until their cervix was fully dilated.

Although ELA is considered to be the most efficient method for labor pain, individual preferences, ELA contraindications or limited availability, and insufficient epidural analgesia effect may require the use of alternative pain-reliving methods during labor including systemic pharmacologic agents and nonpharmacologic methods.

Various nonpharmacologic methods for pain alleviation during labor have become popular over the years, either as a complement to pharmacologic agents or at times as the principal therapy. Methods such as relaxation techniques (Lamaze breathing, yoga, hypnosis, and music) (33), manual techniques (massage, reflexology, and shiatsu) (34), acupuncture (35, 36), birthing ball (37), transcutaneous electrical nerve stimulation (38), and emotional support (doula) (39, 40), are considered safe, although the evidence supporting their effectiveness for pain relief is not as robust as it is for pharmacologic agents.

Systemic pharmacologic agents are mostly administered by inhalation (nitrous oxide) (41) or through the parenteral route. Various opioids such as meperidine, nalbuphine, tramadol, butorphanol, morphine, and remifentanil, mainly exert analgesic effects by acting on opioid receptors in the central nervous system. Acetaminophen provides modest pain reduction compared with placebo and similar effectiveness compared with IV opioids (42, 43), with fewer maternal adverse effects and no need for special monitoring.

Originally, we thought that subjective rejection of ELA was the main reason for not using ELA. To our surprise, the data from this study showed that this only accounted for a small proportion of those who did not use ELA, and a large proportion of them required ELA and it was not available. Therefore, our future work will strive to address these individual and organizational factors and improve ELA rates. Additionally, the reason why the mothers in the requested group were not given ELA is complex, and we will explore it in a future study.

## Clinical trial registration

This study was registered at the Chinese Clinical Trial Registry (Chi CTR 2000034625) on July 12, 2020.

## Data availability statement

The original contributions presented in the study are included in the article/supplementary material, further inquiries can be directed to the corresponding author/s.

## Ethics statement

The studies involving humans were approved by the ethics committee of the Shanghai First Maternity and Infant Hospital. Written informed consent was obtained from the [individual(s) AND/OR minor(s)’ legal guardian/next of kin] for the publication of any potentially identifiable images or data included in this article. The studies were conducted in accordance with the local legislation and institutional requirements. The participants provided their written informed consent to participate in this study.

## Author contributions

WL: Conceptualization, Data curation, Formal analysis, Investigation, Methodology, Project administration, Software, Supervision, Validation, Visualization, Writing – original draft. NW: Conceptualization, Data curation, Formal analysis, Methodology, Project administration, Software, Supervision, Validation, Visualization, Writing – original draft. SZ: Methodology, Project administration, Supervision, Validation, Writing – review & editing. WD: Methodology, Project administration, Supervision, Validation, Writing – review & editing. ZX: Validation, Visualization, Supervision, Writing – review & editing. ZL: Validation, Visualization, Supervision, Writing – review & editing.
