# Supplementary material for: Factors influencing the use of epidural labor analgesia: a cross-sectional survey analysis
Source: Front Med (Lausanne). 2024 Feb 7;10:1280342. doi: 10.3389/fmed.2023.1280342 (PMC10880097; doi:10.3389/fmed.2023.1280342)
Supplement: Supplementary file 2 [file Data_Sheet_2.pdf]

Table 1 Demographic data of Parturients

High (cm)		Weigh (kg)	
BMI		Year	
Primipara (0Nulliparas, 1Multiparas)		Previous vaginal delivery(1F,2T)	
Permanent residence in Shanghai (1F, 2T)		Education	(1Middle school or less, 2High school，3Junior college，4College，5Postgraduate)
Nations (1Han,2Minority)		Religion	(0None,1Buddhism,2Christianity,3islamism)
Diagnoses			

Table 2 Investigation on Cognition of Epidural Delivery Analgesia and Related Factors of Not Performing Epidural Analgesia.

A．The following is a survey on the level of understanding of labor analgesia related knowledge. Please judge if each item is correct.

Questions	Right	Wrong	Unknown
1. ELA would slow the progression of labor			
2. ELA would result in low back pain			
3. ELA would damage nervous system			
4. ELA would impair intelligence			
5. ELA would impair memory			
6. ELA would impair new-born breastfeeding			
7. ELA would impair newborn’s intelligence			
8. ELA can provide analgesia or anesthesia for artificial dissection of the placenta, perineal laceration suture, shoulder dystocia, and hematomatotomy			

B. What level of pain did you experience during childbirth? Please choose a number from 0 to 10 based on your personal experience to rate the pain. (0 No pain, 1-3 Milde pain, 4-6 Modest pain, 7-10 Severe pain).

Questions	Score
1. The pain grade when cervical dilated to 2 cm	
2. The pain grade when cervical fully dilatation	
3. The pain grade of separation of placenta	
4. The pain grade when sew up laceration of perineum	

C The following is a survey on your knowledge of epidural labor analgesia, alternative analgesia,

your attitude towards labor pain, and the reasons for not using epidural analgesia. Please fill in according to your actual situation.

1	Do you require epidural labor analgesia?
	A No, B Yes
2	The reason why you did not undergo epidural labor analgesia delivery (multiple choices available)
	A mild to moderate pain, able to tolerate, B severe pain, able to tolerate, C emergency labor, D charge of epidural labor analgesia
	E husband or other family members refuse, F anesthesiologist during surgery, G Concerns about epidural labor analgesia
	H other reasons:
3	Have you received the epidural labor analgesia brochure from the anesthesia department？
	A No, B Yes
4	What are the ways you can learn about epidural labor analgesia? (multiple choices available)
	A Network, B TV programme, C Relatives and friends, D Books and newspapers, E Child education
	F Midwive ang nurses, G Obstetrcian, H Once ELA
	I Not familiar
5	What are the methods you know to alleviate labor pain？(multiple choices available)
	A Lamaze breathing, B Nitrous oxide, C epidural labor analgesia, DDoula
	E birth in water, F Music, G Transcutaneous Electrical Nerve Stimulation
	H Morphine, Dolantin, I Intravenous analgesia with remifentanil
6	Did you ask for assistance from Doula during your delivery process？
	A No, B Yes
7	Your attitudes towards labor pain？
	A Calm，B Nervous，C Fearful
8	Will you choose ELA for the next natural labor delivery？
	A No，B Yes，C Uncertain

Table 3 Data of birth process

First stage	h	min	Rupture of membrane	Natural / Artificial
Second stage	h	min	Placental delivery method	Natural / Artificial
Third stage	h	min	Position of fetus	
Total stage	h	min	Amount of bleeding	
Instrumental midwifery	1be equal in output, 2episiotomy, 3Forceps Delivery		Degree of perineal tear	0 / I / II / III / IV
Pre amniotic fluid	clear，I，II，III，bloody		Post amniotic fluid	clear，I，II，III，bloody
Episiotomy	A None, B left, C right, D Vaginal tear, E Cervical tear			

Induced abortion method	A Oxytocin， B Artificial rupture of membranes， C Foly balloon， D COOK balloon， E Propess
Surgical name	
Other diagnoses	A None ，B Intrapartum Fever，C postpartum hemorrhage，D hematoma，E Shoulder dystocia，F Placental and fetal membrane defect，G premature birth，H Amniotic fluid embolism， I HELLP ，J Diabetic ketoacidosis，K Coagulation disorders ，L Thromboembolism，M Eclampsia，

Table 4 Data of Newborn

Apgar		Neonatal asphyxia	None / mild / Severe
Long (cm)		Weight (g)	
Gender		Gestational week	
Admission to ICU	A No, B Yes	Reasons	

Table 5 The timeline of your admission and delivery process

	Year	Month	Day	Hour	Min
Onset of labor					
Time of Cervical full dilation					
Time of fetal delivery					
Time of placenta delivery					
Time of rupture					
Date of admit to Emergency					
Time of enter to the ward					
Time of delivery room					
